# Research on Wet Etching Techniques for GaInAs/AlInAs/InP Superlattices in Quantum Cascade Laser Fabrication

**DOI:** 10.3390/nano15050408

**Published:** 2025-03-06

**Authors:** Shiya Zhang, Lianqing Zhu, Han Jia, Bingfeng Liu, Jintao Cui, Tuo Chen, Mingyu Li

**Affiliations:** 1 Department of Optical Engineering, School of Opto-Electronic Engineering, Changchun University of Science and Technology, Changchun 130022, China; 2021200066@mails.cust.edu.cn (S.Z.); chentuo@cust.edu.cn (T.C.); 2 Key Laboratory of the Ministry of Education for Optoelectronic Measurement Technology and Instrument, Beijing Information Science and Technology University, Beijing 100015, China; 2022020220@bistu.edu.cn (H.J.); bingfengliu@mail.hfut.edu.cn (B.L.); 2022020205@bistu.edu.cn (J.C.)

**Keywords:** quantum cascade laser (QCL), wet etching, InP/GaInAs/AlInAs, solution

## Abstract

Wet etching is the mainstream fabrication method for single-bar quantum cascade lasers (QCLs). Different etching solutions result in varying etching effects on III-V semiconductor materials. In this study, an efficient and nearly ideal etching solution ratio was proposed for simultaneously etching both InP and GaInAs/AlInAs, and the surface chemical reactions induced by each component of the etching solution during the process were investigated. Using univariate and single-component experiments, coupled with various characterization techniques such as atomic force microscopy (AFM), stylus profilometer, X-ray photoelectron spectroscopy (XPS), and scanning electron microscopy (SEM), we found that the ratio of HBr to hydrogen peroxide significantly determines the etching rate, while the ratio of HCl to hydrogen peroxide affects the interface roughness. The aim of this study was to provide a comprehensive understanding of the effects of different etching solution components, thereby enhancing the understanding of the wet etching process for InP/GaInAs/AlInAs materials. These findings offer valuable insights into efficient QCL fabrication processes and contribute to the advancement of the field.

## 1. Introduction

Quantum cascade lasers (QCLs), with their unique band structure design and intersubband transitions, have become key light sources in the mid-infrared (MIR) to terahertz (THz) spectral regions [1,2,3]. Their small size, potential for integration, narrow linewidth, and tunable wavelength make them indispensable in applications requiring precise molecular fingerprint detection [4,5,6]. However, challenges remain in achieving efficient operation with high output power and low threshold current density at room temperature [7,8]. The growth of QCL materials based on InP/GaInAs/AlInAs relies on molecular beam epitaxy (MBE) and metal–organic chemical vapor deposition (MOCVD), which, to some extent, can achieve material flattening during epitaxial growth. The precision and quality of the fabrication process are critical to the overall light emission efficiency. In QCLs, the ridge waveguide structure confines light through raised strips (ridges) etched directly on the epitaxial wafer, using the refractive index contrast for lateral optical confinement. It enables efficient mode control in semiconductor lasers. The ridge waveguide structure of a quantum cascade laser can be fabricated using either dry etching or wet etching methods. Bewley et al. found that ridge waveguides fabricated using dry etching [9] may suffer from surface crystal damage, rough sidewalls, surface recombination, and current leakage, leading to higher threshold currents for the QCL. In contrast, ridge waveguides fabricated using wet etching yield smooth sidewalls.

In recent years, significant interest has been generated in the study of etching solutions for different systems [10,11,12,13,14,15]. However, challenges still exist, including unclear experimental mechanisms, weak etching modes, and a lack of understanding of the role of the etching solution components. Furthermore, many descriptions of wet etching processes are overly general and inconsistent. Among the previously reported etching systems for InP/GaInAs/AlInAs materials, commonly used etching solutions include HCl:HNO_3_:H_2_O, HBr:HNO_3_:H_2_O, and HBr:HCl:H_2_O_2_:H_2_O. In the hydrochloric acid system, the HCl + HNO_3_ + H_2_O etching solution has a strong oxidizing property, which is too aggressive for the AZ5214 (a positive-tone photoresist, MicroChemicals GmbH, Ulm, Germany) photoresist used as a mask, leading to the collapse and dissolution of the photoresist during the etching process. Additionally, the surface morphology after etching is poor, making it unsuitable for device fabrication [16]. The hydrogen bromide-based etching solution does not corrode the AZ5214 photoresist, and the HBr + HNO_3_ + H_2_O solution produces a good surface morphology. However, it exhibits strong crystal orientation selectivity, requiring close monitoring of wafer orientation, which makes certain slanted cavity structures impractical [17]. In addition, this solution is typically used to etch the GaInAs/AlInAs active region and needs to be combined with an H_3_PO_4_ + HCl solution to etch the InP upper waveguide layer [18,19]. Moreover, this solution requires at least 24 h of standing time after preparation, and HBr needs to be stored in the dark, which complicates the process and reduces device fabrication efficiency [20,21]. In contrast, the HBr + HCl + H_2_O_2_ + H_2_O etching solution neither corrodes the AZ5214 photoresist nor requires prolonged preparation time, while also enabling the simultaneous etching of InP/GaInAs/AlInAs in a single step.

This study comprehensively evaluates the optimal composition of etching solutions suitable for the InP/GaInAs/AlInAs system, based on HBr:HCl:H_2_O_2_:H_2_O etching liquids, considering factors such as etching rate, surface roughness, and crystal orientation selectivity. The research delves into the role of different etching solution components in the etching process of various materials. This is of significant importance for a deeper understanding of the wet chemical etching characteristics of InP/GaInAs/AlInAs superlattice materials. The study investigates how changes in the etching solution composition can optimize the etching process, enabling precise control over the etching rate and surface roughness. These findings provide valuable guidance for the process fabrication of quantum cascade lasers in this system. Furthermore, examining the effects of each etching solution on different materials will contribute to a more comprehensive understanding of the interactions and properties between materials.

## 2. Materials and Methods

**Figure 1:**
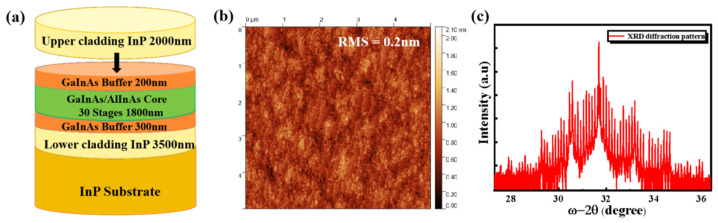
(**a**) Schematic diagram of the etched sample QCL full structure. (**b**) AFM characterization image of the sample surface. (**c**) XRD analysis of the sample structure.

The samples used in this study are QCL full structures, as shown in Figure 1a. All samples were grown on InP (100) substrates via solid-source molecular beam epitaxy (MBE). The growth process began at 440 °C with the deposition of a 300 nm thick GaInAs buffer layer following substrate deoxidation. Subsequently, the substrate temperature was lowered to 420 °C to grow the QCL active core region. The active core consists mainly of Ga_0.31_In_0.69_As/Al_0.64_In_0.36_As quantum wells paired with Ga_0.47_In_0.53_As/Al_0.48_In_0.52_As superlattices, with an overall thickness of approximately 1.8 μm. After the core region growth, a 180–200 nm thick GaInAs buffer layer was deposited to mitigate the effects of secondary epitaxy on the core region. The thickness of the upper waveguide layer of InP is around 2 μm.

The surface defects and roughness of the epitaxial wafer were characterized using a Park NX10 atomic force microscope (AFM), Park Systems, Seongnam, Republic of Korea. As shown in Figure 1b, the sample surface exhibits excellent properties with a roughness of only 0.2 nm. The structural quality of the epitaxial layers was evaluated using a PANalytical X’Pert^3^ MRD high-resolution X-ray diffraction (HRXRD) system, PANalytical, Almelo, The Netherlands. Figure 1c presents the X-ray diffraction spectrum of the structure. The satellite peaks are clearly defined, exhibiting regular satellite peak spacing and a narrow full width at half maximum, with the 0th-order peak coinciding with the substrate peak. These features collectively indicate that the sample possesses outstanding crystal quality, resulting in a flat and strain-balanced surface, providing a solid foundation for subsequent process fabrication.

The etching experiments were conducted in a class 100 cleanroom, with the solution temperature maintained at a constant 25 °C. Additionally, N_2_ drying was employed to suppress surface oxidation and maintain uniform surface conditions. To investigate the effects of wet etching on the various material systems within the structure, we selected HBr (41%), HCl (45%), H_2_O_2_ (30%), and deionized water for the experiments (Table 1). After thoroughly mixing the etching solutions, the mixtures were allowed to sit for one hour until their color changed from transparent to orange-red, allowing the Br- in HBr to be fully oxidized to Br_2_ before the experiment was initiated. To study the effects of each solution on the materials, etching was carried out using several different solutions, including BCHD (a mixture of hydrogen peroxide, HBr, HCl, and deionized water), BHD (a mixture of HBr, H_2_O_2_, and deionized water), CHD (a mixture of HCl, H_2_O_2_, and deionized water), and BCD (a mixture of HBr, HCl, and deionized water).

The etching rates and surface roughness were accurately measured using a KLA Tencor P-17 desktop stylus profiler (KLA Tencor, Milpitas, CA, USA) with a calibrated stylus force of 0.5 mN and a scanning speed of 0.1 mm/s to minimize surface deformation and a non-contact mode of AFM. The solution compositions that resulted in similar etching rates for InP and GaInAs/AlInAs, along with lower surface roughness, were identified. The chemical states of the surface oxide layers for different etching solution compositions were studied using Scienta Omicron X-ray photoelectron spectroscopy (XPS), Scienta Omicron, Ulm, Germany. XPS measurements were conducted in a high-vacuum chamber equipped with an XPS system, which includes an Al X-ray source and a hemispherical electron energy analyzer (EA125), Scienta Omicron, Ulm, Germany. During the experiment, the electron emission angle was set to 63°, and the electron spot size on the X-ray anode was measured to be 1.5 mm × 2.5 mm. During the analysis, an Omicron system FS40, Omicron Nanotechnology, Darmstadt, Germany (charge neutralizer) was employed to compensate for any electron charging effects. The operating parameters of the charge neutralizer were: working energy 1 V, emission current 100 μA, and focusing voltage 300 V. The XPS peaks were calibrated by aligning the C 1 s peak at 284.8 eV. The interface of the samples after etching was characterized using the integrated SEM in a Raith Pioneer two-electron beam lithography system (Raith, Werl, Germany) to evaluate the impact of the etching solution on the crystal orientation selectivity of the sample.

## 3. Results

### 3.1. The Role of Each Component in the BCHD Solution

**Figure 2:**
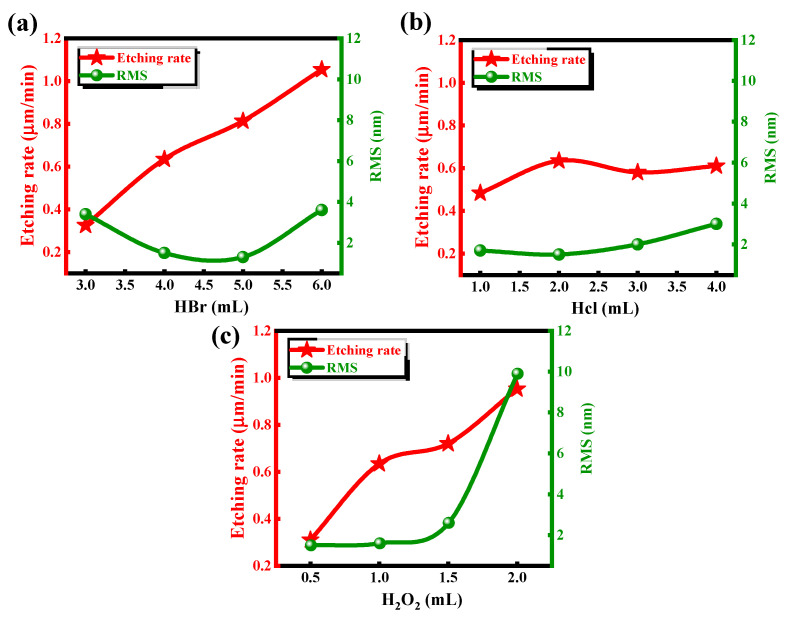
The effects of different contents of three liquids on corrosion rate and roughness correspond to (**a**) HBr (**b**) HCl (**c**) H_2_O_2_.

To investigate the effects of different components on the etching rate and surface roughness, the samples were treated with BCHD solutions of varying concentrations. The initial solution composition was set as 4 mL of HBr, 2 mL of HCl, 1 mL of H_2_O_2_, and 50 mL of DIW. Keeping other solution components constant, univariate experiments were carefully conducted by independently varying the concentrations of hydrogen bromide, hydrochloric acid, and hydrogen peroxide in each solution over discrete time intervals. Each experiment was repeated three times under identical conditions. Figure 2 reported etch rates and roughness values represent mean ± standard deviation, with variations below 6% between trials.

As shown in Figure 2a,c, it is clear that the etching rate is strongly positively correlated with the concentrations of HBr and H_2_O_2_. As the concentration of HBr increases, the etching rate rises sharply. In contrast, the effect of HCl on the etching rate is relatively minimal; even when the concentration of HCl increases by a factor of four, the etching rate remains around 0.6 μm/min, as shown in Figure 2b. The concentration of H_2_O_2_ also significantly affects the etching rate; when the concentration is 1 mL or 1.5 mL, the curve stabilizes, and the etching rates are nearly identical. At concentrations of 0.5 mL and 2 mL, the etching rate shows a sharp decrease and increase, respectively. To achieve the best etching results with a smooth bottom surface and interface, AFM measurements were performed on the bottom surfaces of 12 groups of samples after etching and de-photoresist processing. We found that excessive H_2_O_2_ causes the bottom surface roughness to increase dramatically to 10 nm, which significantly affects the material quality. The effect of HCl showed a slight increasing trend in roughness with increasing concentration, but even when the concentration reached 4 mL, the roughness was only 3.2 nm. The relationship between HBr concentration and bottom surface roughness is nonlinear. The least impact on the bottom surface roughness occurred at concentrations of 4 mL and 5 mL, while at concentrations of 3 mL and 6 mL, the roughness was around 4 nm.

The wet etching process mainly consists of oxidation and decomposition. As shown in Equation (1), in the BCHD solution, the orange-red solution formed after mixing and standing contains Br_2_ as the core oxidizing agent. H_2_O_2_ assists in the oxidation of Br^−^ and participates in some of the oxidation reactions. Therefore, an excess of H_2_O_2_ and HBr leads to an oxidation rate that exceeds the dissolution rate, causing the accumulation of insoluble substances, which results in surface roughness [22]. Increased HBr concentration directly increases the Br_2_ concentration, significantly accelerating the oxidation reaction rate. As a result, increases in both HBr and H_2_O_2_ concentrations lead to a noticeable increase in the etching rate. In contrast, the role of HCl in the etching solution is primarily to provide H⁺ ions, maintaining a strongly acidic environment. We speculate that, under such acidic conditions, the oxidation capability of H_2_O_2_ may not be sufficient to oxidize Cl^−^, while it is more likely to oxidize Br^−^ due to the lower oxidation potential of Br^−^. Therefore, Cl^−^ may primarily act as a ligand rather than being oxidized. The main role of Cl^−^ is to form soluble complexes with metal ions generated during oxidation (such as In^3+^, Ga^3+^, and Al^3+^), preventing the deposition of insoluble oxides, thereby facilitating their dissolution. The coordinating effect of Cl^−^ on the etching rate is indirect and mainly manifests in the dissolution of oxidation products and maintaining the uniformity of the etching solution. Considering the similar etching rates for InP and GaInAs/AlInAs materials, as well as the roughness of the bottom surface, the optimal BCHD solution composition for wet etching was chosen to be 5:2:1:50.

H_2_O_2_ + 2H^+^ + 2Br^−^ → 2H_2_O + Br_2_ (1)

### 3.2. Morphological Changes After Etching with Each Solution

**Figure 3:**
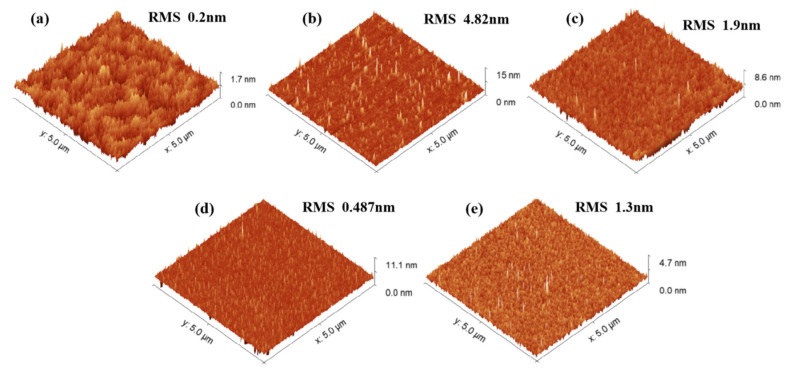
The figure shows 5 µm × 5 µm AFM images. (**a**) Untreated sample (**b**) Sample after etching with BHD solution. (**c**) Sample after etching with CHD solution. (**d**) Sample after etching with BCD solution. (**e**) Sample after etching with BCHD solution.

Further analysis of the significant effects brought about by the optimized ratio is crucial to fully understand the individual contribution of each component in the etching solution to the overall result. To further verify the role and impact of each component in the etching solution, we conducted multiple experiments under different conditions. Figure 3 compares the surface morphology of untreated and selectively treated surfaces, with the same experimental time for each condition. Figure 3a shows the morphology of the untreated original surface, which is very smooth, exhibiting atomic step-like features. Figure 3b presents the surface morphology after etching with the BHD solution, where numerous protruding defects and high roughness are observed. The etching rate is about 0.52 μm/min, indicating that a single HBr combined with H_2_O_2_ can still effectively corrode the sample. However, the absence of Cl^−^ in the solution prevents the formation of coordinated soluble complexes. As a result, Br^−^ has weak coordination ability, leading to the deposition of metal ions in the form of hydroxides/oxides on the surface. These insoluble substances hinder the uniformity of the etching solution, thereby increasing the surface roughness. In contrast, the corrosion effect of the CHD solution, which does not include HBr, is shown in Figure 3c. The surface roughness decreases to 1.9 nm, and the needle-like protrusions are significantly reduced, with the etching rate being very slow at only 1 nm/min. This is because the oxidation potential of Br_2_ (~1.07 V) is significantly higher than the effective oxidation potential of H_2_O_2_ (~0.7 V) in acidic conditions, allowing it to more efficiently oxidize elements such as In, Ga, Al, P, and As in the material and disrupt their lattice structures [23,24]. In the CHD system, H_2_O_2_ cannot effectively replace the oxidation function of Br_2_, leading to surface passivation and preventing the full initiation of the Cl^−^ dissolution process. Figure 3d shows the BCD solution, which exhibits a similar effect to the CHD system. Due to the absence of H_2_O_2_, neither HBr nor HCl undergoes oxidation, and the oxidation and dissolution processes do not occur, leading to almost no etching effect on the surface, with a corrosion rate of only 0.2 nm/min. Figure 3e presents the etching morphology when the BCHD solution is mixed at a ratio of 5:2:1:50. The sample shows only minor defects, but the surface remains overall flat with a roughness of just 1.3 nm. The corrosion rate is an ideal 0.85 μm/min.

### 3.3. Surface Properties of InP and GaInAs/AlInAs

**Figure 4:**
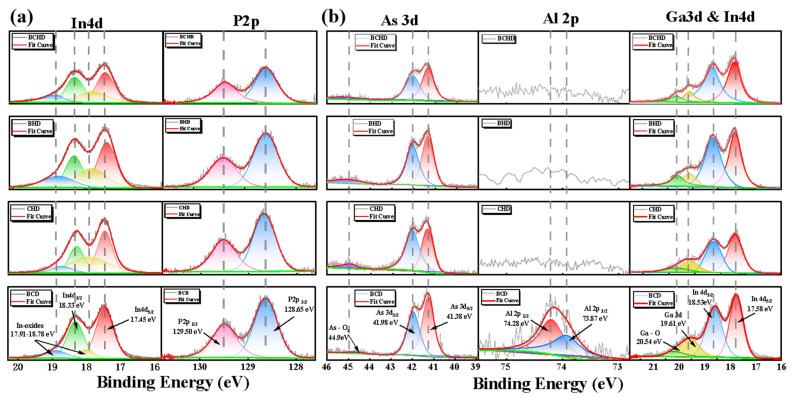
XPS spectra of samples were etched for 2 min with BCHD, BHD, CHD, and BCD solutions. (**a**) InP (100) surface showing In 4d and P 2p peaks. (**b**) GaInAs/AlInAs superlattice showing As 3d, Ga 3d and In 4d, as well as Al 2p peaks.

To further investigate the changes in material composition during the etching process of this material system, we conducted separate experiments on the undoped QCL waveguide structure (InP (100)) and the QCL core region structure (GaInAs/AlInAs superlattice). The chemical states of the surface treatment of these elements were quantitatively analyzed using XPS in different solutions, including the four solutions mentioned above. The XPS spectra were fitted using a least squares fitting program. To fit specific core-level peak values, a model function consisting of convolution Gaussian and Lorentzian functions was employed. Additionally, Shirley’s background subtraction was used to effectively remove background noise. The results are shown in Figure 4. After wet etching InP, split peaks of P 2p were found at 129.5 eV and 128.65 eV. In 4d, peaks were observed at 17.45 eV and 18.33 eV, while In-O peaks appeared at 17.91 eV and 18.78 eV. As seen in Figure 4a and Table 2, the absence of H_2_O_2_ in the BCD solution resulted in ineffective oxidation of InP, leading to a low In-O content. In contrast, both Br_2_ and H_2_O_2_ in the CHD and BHD solutions effectively oxidized InP, causing an increase in the relative In-O content. Under CHD treatment, the overall In-P content was lower than that under BHD, which may be due to the fact that In is more dependent on Cl^−^ coordination compared to Br^−^. In the BCHD solution, perfect oxidation and dissolution occurred, resulting in a significant decrease in the overall intensity of In-P elements. The possible oxidation and dissolution reactions that may occur with InP are detailed in [1]:

2In + 3Br_2_ → 2In^3+^ + 6Br^−^/2In + 3H_2_O_2_ → 2In^3+^ + 6OH^−^
In^3+^ + 4Cl^−^ → [InCl_4_]^−^
4P + 5Br_2_ → 2P_2_O_5_ + 10Br^−^/4P + 5H_2_O_2_ → 2P_2_O_5_ + 5H_2_O P_2_O_5_ + 3H_2_O → 2H_3_PO_4_ (2)

Among them, both H_2_O_2_ and Br_2_ may produce oxidation reactions on In and P. The oxidized In element is In^3+^, which mainly forms a soluble complex with Cl^−^, and the P element is dissolved in water in the form of phosphoric acid, causing P^5+^ to detach from the surface.

In the GaInAs/AlInAs material, As exists in the As^3−^ valence state, bonding with Ga, Al, and In. Split peaks of As 3d were measured at 41.38 eV and 41.98 eV and an As-O peak was detected at 44.9 eV. During the etching process, As^3−^ can be oxidized by Br_2_ or H_2_O_2_ to As^5+^, resulting in the formation of arsenic oxides (such as As_2_O_5_) or arsenic acid (H_3_AsO_4_), which dissolves in water.

2As + 5Br_2_ → 2As_2_O_5_ + 10Br^−^/2As + 5H_2_O_2_ → As_2_O_5_ + 5H_2_O  As_2_O_5_ + 3H_2_O → 2H_3_AsO_4_ (3)

As shown in Table 2, a large amount of arsenic oxide was generated under the influence of BHD and CHD; however, there was almost no presence of As-O in BCHD and BCD. This might be because BCD failed to oxidize As, while the combination of HBr and HCl with hydrogen peroxide caused localized pH changes or incomplete oxidation, leaving traces of As_2_O_5_ or other intermediate oxides, resulting in the appearance of the As-O peak. The complete fusion in BCHD enhanced the oxidation and hydrolysis of As-O.

The changes in the group III metal elements are also reflected in Figure 4b. Due to the high susceptibility of Al to oxidation, a relatively high Al-O peak is observed even in the untreated sample and under the effect of BCD. In CHD, BCD, and BCHD, the presence of Cl^−^ and Br^−^ enhances the reactivity of Al, allowing it to quickly form complexes and dissolve, which results in no detectable Al content. The Ga-O content gradually increases under the effect of CHD and BHD, and after complete oxidation in BCHD, Ga dissolves thoroughly, with the Ga-As content reduced to half that observed under BCD. In contrast, it was found that the Ga-As content under BHD treatment is significantly lower than that under CHD. This may be because Br_2_ fully oxidizes Ga, and Br^−^ also readily forms complexes with Ga^3^⁺. This phenomenon is opposite to the effect observed for the In element in InP.

Ga^3+^/Al^3+^ + 4Cl^−^ → [Ga/AlCl_4_]^−^
 Ga^3+^/Al^3+^ + 4Br^−^ → [Ga/AlBr_4_]^−^ (4)

By synthesizing the corrosion effects of the four etching solutions on the two material systems, we found that Br_2_ in HBr has a strong oxidizing effect and is the primary source of oxidation for the materials. Additionally, Br^−^, as a halide ion, can form soluble complexes with metal ions, although its complexing ability is lower than that of Cl^−^. Among the elements, Al and Ga are more sensitive to Br^−^ compared to In, which requires Cl^−^ coordination for more effective dissolution. In other words, HBr primarily works with H_2_O_2_ to exert an oxidizing effect, while HCl mainly enhances the dissolution process. Under the combined action of HBr and HCl, the oxides of non-metallic elements, such as As and P, are more efficiently hydrolyzed into acids, which dissolve in water. This results in reduced surface roughness, increased overall uniformity, and improved smoothness of the samples.

### 3.4. Crystal Orientation Selectivity of the BCHD Etching Solution

**Figure 5:**
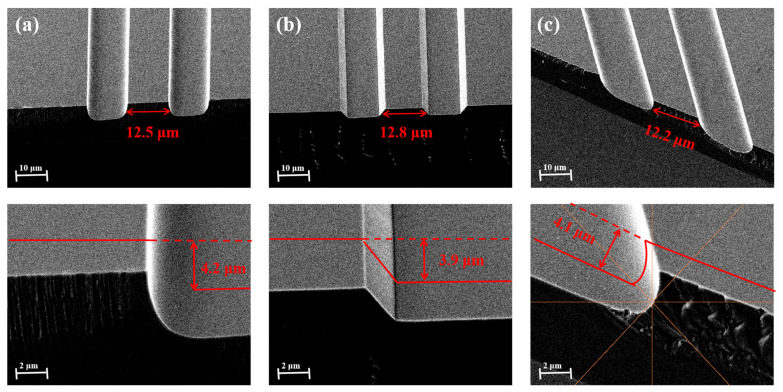
Ridge profile after BCHD etching. (**a**) Perpendicular to the wafer major flat. (**b**) Parallel to the wafer major flat. (**c**) Tilted at a 45-degree angle to the major flat.

To investigate whether the BCHD etching solution affects the crystal orientation selectivity of the epitaxial wafer, we performed wet etching along the perpendicular, parallel, and 45° tilted orientation relative to the substrate’s off-angle orientation. As shown in Figure 5a, for the etching perpendicular to the wafer direction, the etching depth is 4.2 μm, with the active region width being 12.5 μm. The sidewalls exhibit smoothness and uniformity. In the parallel direction (Figure 5b) and at a 45° tilt (Figure 5c), the active region widths are 12.8 μm and 12.2 μm, respectively, accompanied by sub-micron lateral undercutting. Relatively, etching morphologies along the vertical wafer direction and at a 45° angle to the wafer are more suitable for process fabrication. The slight lateral etching results in a smaller cross-sectional area of the active region compared to the upper waveguide layer. For electrically injected quantum cascade lasers (QCLs), this can concentrate electron injection, thereby improving electron injection efficiency to some extent.

It can be observed that this etching solution still exhibits slight crystal orientation selectivity, as the etching profile depends on the ridge direction relative to the wafer plane. In other words, the etching is not exactly isotropic, but the lateral undercutting in all three directions is very small, suggesting that precise tracking of the wafer orientation during etching becomes less critical. This means that the BCHD wet etching process can continue to be used in the subsequent manufacture of lasers with special structures such as ridge-shaped bevel cavities that can amplify pulse power.

## 4. Discussion and Conclusions

This study employed a univariate experimental approach to investigate the effects and influence of each component in the wet etching solution on the InP/GaInAs/AlInAs material system. Our results show that, when the HBr:HCl:H_2_O_2_:H_2_O = 5:2:1:50, a uniform etching rate of 0.85 μm/min can be achieved for InP/GaInAs/AlInAs, with a post-etch surface roughness of only 1.3 nm. This composition not only allows precise control over the etching rate but also results in a smooth etching effect. Through univariate adjustments during this process, we identified that HBr and H_2_O_2_ are the key factors controlling the etching rate, and effective modulation of HCl and H_2_O_2_ can reduce the roughness of the post-etch interface. It is worth noting that the uniformity of the etching rate is somewhat dependent on strict control over the ambient temperature (25 ± 0.5 °C) and standardized surface pretreatment, which involves sequential cleaning with acetone, anhydrous ethanol, and deionized water. Thermal drift can induce variations in the etching rate due to changes in reaction kinetics, while residual organic contaminants (thickness > 3 nm) may locally inhibit halide coordination, thereby exacerbating surface roughness.

By controlling the presence of individual solution components and combining AFM and XPS analyses, we found that during the solution preparation process, Br_2_, which is oxidized from HBr by H_2_O_2_, exerts a strong oxidative effect on III–V materials. Meanwhile, Br^−^ and Cl^−^ from HCl, as halides, can form water-soluble complexes with metals in Group III. Cl^−^ shows a strong affinity for Ga, Al, and In, while Br^−^ readily forms complexes with Al and Ga but is less likely to form complexes with In. For Group V elements, both H_2_O_2_ and HBr alone can induce oxidation; however, due to pH effects, the oxides may not fully dissolve. When HCl is added, complete dissolution can be achieved. In summary, the HBr:HCl:H_2_O_2_:H_2_O etching solution can efficiently etch GaInAs/AlInAs and effectively etch InP. Compared to traditional multi-step processes (e.g., etching InP with H_3_PO_4_:HCl followed by switching to HBr:HNO_3_:H_2_O for GaInAs/AlInAs treatment), this single-step method significantly enhances process efficiency and eliminates the risk of interfacial oxidation inherent in multi-step approaches. However, the etching rate is highly sensitive to mixture parameters; minor variations in HBr and H_2_O_2_ concentrations can substantially alter the etching rate. Thus, strict control of the mixture ratio and pH is required to ensure reproducibility, tailored to the specific materials. The optimized single-step process demonstrates notable advantages in simplifying fabrication and defect control, making it particularly suitable for high-density III–V heterojunction device manufacturing.

Considering the practical process requirements and the need for resonant cavity structure fabrication, the etching solution was tested for crystal orientation selectivity. It was found that the etching solution exhibits some degree of crystal orientation selectivity; however, the resulting beveling of the bottom surface and lateral etching are relatively weak. Etching perpendicular to the wafer direction or at a 45° angle to the wafer both produce excellent cross-sectional results. This indicates that the solution not only simplifies the process but also enhances the practical applicability for the fabrication of various simple waveguide structures.

## Figures and Tables

**Table 1:** Abbreviations for the different etching solution compositions designed in this experiment.

	HBr (Hydrobromic Acid)	HCL (Hydrochloric Acid)	H_2_O_2_ (Hydrogen Peroxide)	DIW (Deionized Water)
BCHD	Add	Add	Add	Add
BHD	Add	/	Add	Add
CHD	/	Add	Add	Add
BCD	Add	Add	/	Add

**Table 2:** XPS Spectrum analysis of elemental composition on the sample surface.

Peak	Component	Intensity Ratio Versus Bulk			
		BCHD	BHD	CHD	BCD
P 2p	P 2p _3/2_	60.42%	62.47%	62.61%	61.4%
	P 2p _1/2_	39.58%	37.53%	37.39%	38.6%
In 4d	In-P	73.34%	70.13%	68.38%	79.63%
	In-O	26.66%	29.87%	31.62%	20.38%
As 3d	As	92.4%	84.17%	90.34%	96.15%
	As-O	7.6%	15.83%	9.66%	3.85%
Al 2p	Al-As	/	/	/	42.3%
	Al-O	/	/	/	57.7%
Ga3d and In4d	Ga-As	8.43%	8.21%	14.2%	15.5%
	Ga-O	6.28%	9.17%	6.73%	4.05%
	In 4d_5/2_	40.29%	39.6%	42.13%	41.4%
	In 4d_3/2_	45%	43.01%	36.94%	39.05%

## Data Availability

Data underlying the results presented in this paper are not publicly available at this time but may be obtained from the authors upon reasonable request.

## Abbreviations

The following abbreviations are used in this manuscript:

BCHD	Etching solution prepared from a mixture of HBr, HCl, hydrogen peroxide, and deionized water
BHD	Etching solution prepared from a mixture of HBr, hydrogen peroxide, and deionized water
CHD	Etching solution prepared from a mixture of HCl, hydrogen peroxide, and deionized water
BCD	Etching solution prepared from a mixture of HBr, HCl, and deionized water
